# Hemoadsorption Using CytoSorb^®^ in Patients with Infective Endocarditis: A German-Based Budget Impact Analysis

**DOI:** 10.3390/jcdd10090366

**Published:** 2023-08-26

**Authors:** Cristina Rao, Franziska Preissing, Matthias Thielmann, Daniel Wendt, Zaki Haidari, Jurij Matija Kalisnik, Lothar Daake, Karl Traeger

**Affiliations:** 1 CytoSorbents Europe, 12587 Berlin, Germany; 2 Department of Thoracic and Cardiovascular Surgery, West-German Heart & Vascular Center, 45122 Essen, Germanyzaki.haidari@uk-essen.de (Z.H.); 3 Department of Cardiac Surgery, Klinikum Nürnberg, Paracelsus Medical University, 90419 Nuremberg, Germany; 4 Medical Controlling Department, University of Essen, 45157 Essen, Germany; 5 Department of Cardiac Anesthesiology, University Hospital Ulm, 89081 Ulm, Germany

**Keywords:** acute infective endocarditis, extracorporeal techniques, hemoadsorption, cytosorb, cardiopulmonary bypass surgery, economic evaluation

## Abstract

A considerable number of infective endocarditis (IE) patients require cardiac surgery with an increased risk for postoperative sepsis. Intraoperative hemoadsorption may diminish the risk of postoperative hyperinflammation with potential economic implications for intensive care unit (ICU) occupation. The present study aimed to theoretically investigate the budget impact of a reduced length of ICU stay in IE patients treated with intraoperative hemoadsorption in the German healthcare system. Data on ICU occupation were extrapolated from a retrospective study on IE patients treated with hemoadsorption. An Excel-based budget impact model was developed to simulate the patient course over the ICU stay. A base-case scenario without therapy reimbursement and a scenario with full therapy reimbursement were explored. The annual eligible German IE patient population was derived from official German Diagnostic-Related Group (DRG) volume data. One-way deterministic sensitivity analysis and multivariate analysis were performed to evaluate the uncertainty over the model results. The use of intraoperative hemoadsorption resulted in EUR 2298 being saved per patient in the base-case scenario without therapy reimbursement. The savings increased to EUR 3804 per patient in the case of full device-specific reimbursement. Deterministic and probabilistic sensitivity analyses confirmed the robustness of savings, with a probability of savings of 87% and 99% in the base-case and full reimbursement scenario, respectively. Intraoperative hemoadsorption in IE patients might have relevant economic benefits related to reduced ICU stays, resulting in improved resource use. Further evaluations in larger prospective cohorts are warranted.

## 1. Introduction

Despite improvements in standard-of-care management, infective endocarditis (IE), especially in cases of severe valvular dysfunction and perivalvular abscesses, still represents a complex disease with a high risk of severe congestive heart failure and cardiac-related death [1].

Recent advancements in antibiotic therapy have improved the clinical course of IE, however, 25% to 50% of patients with acute IE still require surgery for the replacement or reconstruction of the valves and annular tissue [2]. Mortality rates remain high, with reports of 6% to 50% in-hospital mortality in the worst-case scenario, as well as significantly reduced long-term survival ranging between 18% and 81% at five years [1,3].

Cardiac surgery in patients presenting with IE carries a disproportionately increased risk for peri-operative hyperinflammation and post-operative sepsis due to the dissemination of bacteria from the infected valves, and the release of cytokines and vasoactive peptides triggered by the cardiopulmonary bypass (CPB) itself [4,5,6].

CytoSorb^®^ (CytoSorbents, Princeton, NJ, USA) is a CE-marked whole-blood hemoadsorption device capable of removing hydrophobic low- and medium-sized substances with a molecular weight up to 60 kDa (including cytokines, myoglobin, and bilirubin). Removal is concentration-dependent, implying that substances at high concentrations are removed more effectively than those circulating in blood at lower concentrations. Clinical studies suggest a potential effectiveness in ameliorating inflammation and reducing organ injuries [7,8,9]. The therapy has been increasingly used in patients with hyperinflammation from various etiologies, including septic shock, rhabdomyolysis, liver failure, and severe acute pancreatitis. Recently, beneficial effects have been observed in patients developing perioperative renal failure in combination with severe hemodynamic instability [10].

The therapy can be easily integrated into the CPB circuit for intraoperative use during cardiac surgery and is indicated in patients with infective endocarditis to reduce proinflammatory mediators [11].

In addition to the various clinical benefits observed by different studies on intraoperative use in IE patients [7,12,13,14,15], CytoSorb^®^ adjunctive therapy might have relevant economic implications derived from the reduced perioperative need for vasoactive drugs and from general optimization in resource use. In particular, two studies to date have shown a shorter ICU stay in IE patients treated intraoperatively with CytoSorb^®^, compared to patients undergoing standard surgical treatment [11,13].

Despite this reduction in ICU occupation, it could not be confirmed in other clinical studies [14,15,16,17] and requires further investigation. In this study, we assessed the potential economic impact of a theoretical reduction in ICU length of stay associated with CytoSorb^®^ use in IE patients undergoing cardiac surgery. We adapted the model to the German healthcare system and to its eligible surgical IE population.

## 2. Materials and Methods

A Microsoft^®^ Excel-based [18] budget impact model was developed to estimate the economic impact of adding intraoperative hemoadsorption with (one cartridge of) CytoSorb^®^ to standard surgical treatment of severe IE in Germany. A one-year period was used to estimate the potential population eligible for surgical treatment with adjunctive CytoSorb^®^ therapy in Germany. The patient population was calculated based on official hospital volume data sources (see Section 2.3 Patient Population and Appendix A for details). Only effects deriving from differences in the ICU stay were considered to assess the economic implications of the intraoperative use of the therapy. Deterministic and probabilistic sensitivity analyses were performed to test the robustness of results.

### 2.1. Data on Resource Use

Data on ICU length of stay were extrapolated from a retrospective case series comparing 39 IE patients treated with CytoSorb^®^ hemoadsorption to an historical control group of 28 patients who did not receive intraoperative hemoadsorption [11]. The investigators reported a difference of 2.5 days in ICU length of stay, which was equal to 5 (IQR:4–8) days in the treated group and 7.5 (4.5–10) in the historical controls (*p* = 0.059). This finding has limitations inherently linked to the design and small sample size of the study and to the existence of contradicting results in the literature [7,14,15,16,17]. We nevertheless considered building a theoretical model based on this result, aware that more data and evidence are required to confirm this assumption in a real-world setting.

### 2.2. Cost Data

The therapy cost of using one CytoSorb^®^ adsorber intraoperatively was calculated in accordance with the guidance published by the German Institute for Reimbursement in Hospitals (InEK) [19]. The guidance provides instructions on how to calculate additional reimbursements (“Zusatzentgelt”) for specific procedures in general, and specifically for dialysis and related procedures, as provided in attachment F of the guidance. These methods and calculations also constitute the basis for negotiating an individual reimbursement rate at the hospital level.

In compliance with these requirements, we calculated the therapy costs by taking into consideration the selling price of the device (including priming and CPB adapters) in Germany, adding 19% VAT and the personnel costs (physicians). The latter were estimated based on conservative assumptions on the expected time required for installing (approximately 30 min), monitoring (15 min), and removing the adsorber (15 min). These assumptions were built for a hypothetical hospital where the therapy would not be used routinely. We are aware shorter times are required for the therapy setting in most experienced hospitals. Based on estimates validated by experts from the medical controlling department of one German university hospital involved in this research, the average cost of the medical personnel was set to equal EUR 1 per minute, resulting in personnel costs of EUR 60 in total. Furthermore, in accordance with the InEK cost calculation guidance, an exemplary infrastructure surcharge was considered and added to calculate the full cost of the device [19]. The infrastructure surcharge is set individually by hospitals, therefore, based on feedback from our network, we assumed it to be equal to 12% and applied it to the overall material and personnel costs of the therapy.

The average cost of a stay of one day in ICU was extrapolated from the literature [20] and updated according to the German Health Consumer Price Index to 2022 healthcare prices.

Details of the cost calculations are displayed in Table 1.

### 2.3. Patient Population

The annual patient population of surgical IE patients thought to be eligible for treatment with CytoSorb^®^ was estimated using German diagnosis, procedures, and DRG official volume data for the year 2021. Diagnosis, procedure, and DRG volume data were retrieved from the reimbursement.INFO platform [21], while the reimbursement data were retrieved from the German Institute for Reimbursement in Hospitals, InEK.

Diagnosis and DRG case data were combined with data on the use of the OPS procedure code for hemoadsorption with CytoSorb^®^ (OPS 8-821.2 “Extracorporeal adsorption of low and medium molecular weight hydrophobic substances”) to extrapolate the number of surgical infective endocarditis episodes where CytoSorb^®^ was used.

In 2021, 15,477 patients were diagnosed with infective endocarditis in Germany [21]. These cases were all coded with ICD-10 codes for Infective Endocarditis (I33.0 and I33.9) as primary or secondary diagnoses. They were all grouped in 187 medical and surgical DRGs of different complexity. Four out of the top six most frequent DRGs were surgical DRGs, suggesting that a total of 2489 acute IE patients (19.5%) required heart valve surgery with a heart lung machine (F03A to F03D). Among these DRGs, we selected those reporting on cases where the OPS procedure code 8-821.2 was used (634 cases). In total, 634 cases were found where the procedure code 8-821.2 was coded in surgical DRGs for heart valve surgery in IE patients. There were no other relevant surgical DRGs where the diagnosis codes for infective endocarditis were used in combination with the procedure code for hemoadsorption (see Appendix A and Table A1 for details).

To account for possible overestimates—i.e., due to use of CytoSorb^®^ in the post-operative setting only, or due to coding of other hemoadsorption devices—the number of patients with IE requiring heart valve surgery and receiving CytoSorb hemoadsorption therapy was rounded down to 550 patients.

### 2.4. Scenario Analyses

We considered intraoperative installation of one cartridge of CytoSorb^®^ hemoadsorption therapy into the CPB circuit during cardiac surgery for IE. Two hospital financing scenarios were considered. In the first scenario, the differences in costs between the two therapeutic strategies were calculated assuming that the hospital received no device-specific reimbursement for CytoSorb^®^ therapy; in the second scenario, we assumed that sickness funds would provide full therapy reimbursement for the therapy, equal to the full cost of CytoSorb^®^ as calculated in Table 1. The analysis was conducted over a potential annual population of 550 patients for the whole German hospital system. Results of the economic impact of treating one patient are also presented.

### 2.5. Sensitivity Analyses

Sensitivity analyses were conducted to assess the confidence in the results of the model. A one-way deterministic sensitivity analysis was performed by varying the base-case model inputs one at a time. A variation of ±50% was used for the daily cost of ICU stay, the infrastructure surcharge range was set to a range between 0% and 100% of the therapy cost and a variation of ± for the interquartile range (IQR) was used for the change in ICU length of stay. The ±50% variation in ICU costs was chosen after internal discussion between stakeholders of different hospitals in Germany. The 0% to 100% range in infrastructure surcharge was applied to take into consideration the specificity of this parameter to individual hospitals in Germany. While we acknowledge that a 100% surcharge is unlikely, we included it in the model for the therapy cost for theoretical and conservative reasons and to highlight the large variability that might exist in different hospitals’ administrations. Finally, the upper and lower quartiles for the ICU length of stay were used as a good measure of the variability in the studied parameter. A tornado diagram was used to visually demonstrate the resulting reduction in costs corresponding to the altered model input and to the maximum and minimum values of the range.

To further reduce the uncertainty associated with input parameters, a probabilistic sensitivity analysis was conducted. A bootstrap analysis for the input parameter ICU length of stay was conducted to estimate the difference in the parameter’s value between the treated and control populations. The bootstrap consisted of 1000 samples with 100 observations each and was developed in Microsoft Excel’s visual basic environment [18]. The resulting lengths of stay were multiplied by the daily cost of ICU stay, which was calculated using a two-parameter gamma distribution. The gamma distribution was chosen and used, the reference probability distribution being recommended for the probabilistic assessment of cost variables in cost-effectiveness analysis and health decision modeling [22].

## 3. Results

**Figure 1:**
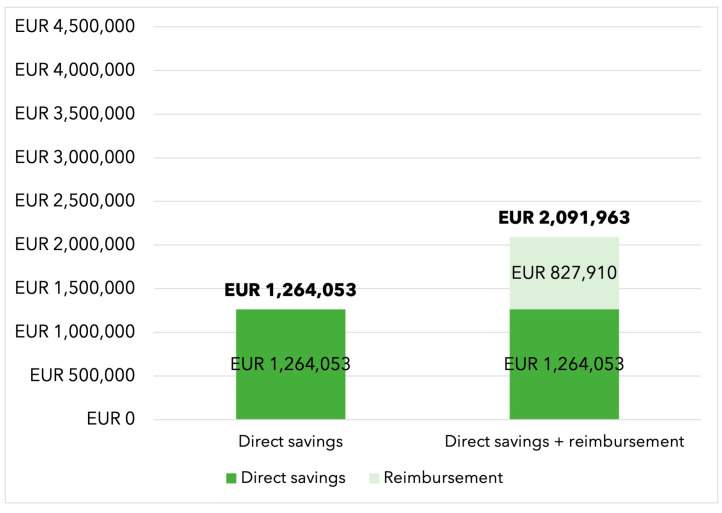
Direct savings and savings from therapy reimbursement over a one-year expected infective endocarditis population.

In the base-case scenario without dedicated reimbursement for hemoadsorption therapy, the additional use of CytoSorb^®^ therapy resulted in total savings of EUR 1,264,053 when treating an annual potential population of 550 cases, corresponding to EUR 2298 saved per patient. The savings increased to EUR 2,091,963 (EUR 3804 per patient) in cases of full therapy reimbursement (see Figure 1).

**Figure 2:**
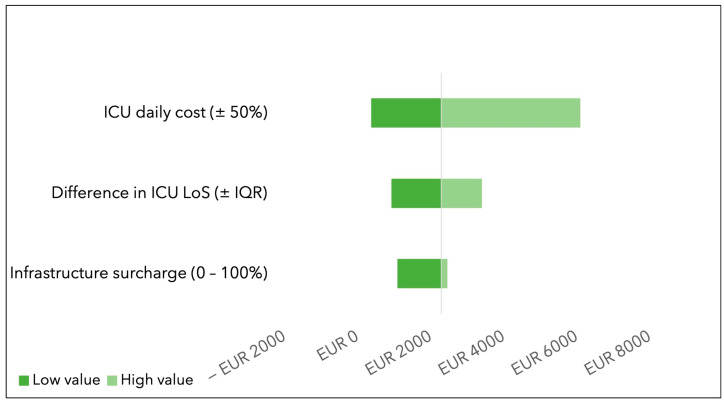
One-way sensitivity analysis for use of CytoSorb^®^ in one infective endocarditis patient.

In the one-way sensitivity analysis, the variable with the highest impact was the cost of ICU stay. When varying the cost of ICU stay by ±50% of its value, the savings ranged from EUR 396 (in the case of a 50% decrease in the cost of an ICU stay) to EUR 6102 (in the case of a 50% increase in the cost of ICU occupation). The variation in infrastructure surcharge had the lowest impact on the range of savings (see Figure 2).

The tornado diagram displays how the savings change when input variables are changed one at a time. A 50% reduction in the ICU daily cost lowers savings to EUR 396, while a +50% variation in the daily ICU costs increases changes to EUR 6102. A decrease in the difference observed in the ICU length of stay between the two groups lowers savings to EUR 950, while an increase in ICU length of stay difference increases savings to EUR 3406. Finally, by increasing the infrastructure surcharge applied to the therapy cost to the full cost of the therapy (100% surcharge), the savings diminish to EUR 1116, while the savings equal EUR 2460 in the case that no infrastructure surcharge is applied by the hospital controlling unit (0%).

A probabilistic sensitivity analysis was conducted using the difference in the bootstrap analysis of the ICU length of stay in the two groups and the probabilistic value of the ICU daily cost (see Sensitivity Analysis in Section 3).

**Figure 3:**
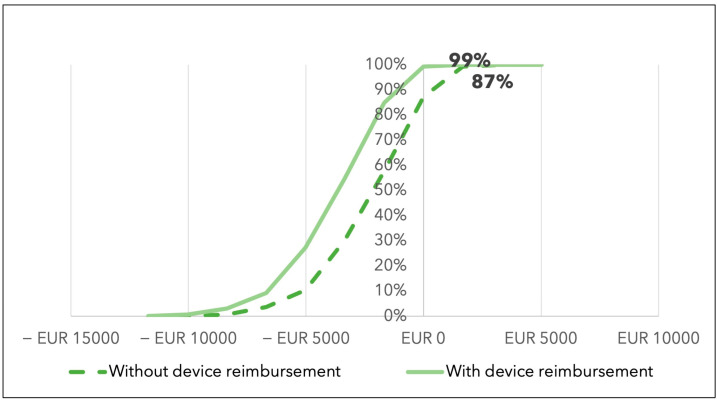
Probability of CytoSorb^®^’s cost saving.

These results suggest that CytoSorb^®^ has a probability of saving costs equal to 87% in the base-case scenario, and to 99% in cases of full therapy reimbursement (see Figure 3).

The two lines indicate the probability of CytoSorb^®^ therapy being cost saving in the two scenarios, without (baseline) and with full therapy reimbursement. The *x*-axis indicates the difference in costs between CytoSorb^®^ and standard therapy, while the *y*-axis reports the different probabilities of savings. The probability of CytoSorb^®^ resulting in a cost difference of at least EUR 0 compared to the standard of care is 87% in the scenario without reimbursement (baseline), increasing to 99% in case of full therapy reimbursement.

## 4. Discussion

This study investigated the economic implications of using intraoperative hemoadsorption with CytoSorb^®^ in high-risk infective endocarditis patients requiring cardiac surgery in Germany. We observed that, even without reimbursement, intraoperative hemoadsorption leads to EUR 1,264,053 (EUR 2298 per patient) cost savings per annum, which increases to EUR 2,091,963 (EUR 3804 per patient) in cases of full therapy reimbursement. Daily ICU costs were the major cost driver in the postoperative period and, even after conducting sensitivity analyses and varying the daily ICU costs by +/−50%, savings were still observed.

Currently, CytoSorb^®^ therapy is coded in Germany through the specific procedure code OPS 8-821.2 ‘Extracorporeal adsorption of low and medium sized molecular, hydrophobic substances (including cytokine adsorption)’. The use of this code is linked to an additional reimbursement (ZE) on top of the DRG rate, which is negotiated every year by hospitals and sickness funds [23], and which was assumed to be in place in the second scenario of this German-specific analysis.

Notably, the findings of the present study can be generalized to other European and non-European health care systems with similar financing and cost structures. Although costs vary across countries and hospitals, and depend ultimately on the medical complexity of treated cases, the average daily costs for ICU occupation are in a similar cost range in European countries, which reflects the availability of homogeneous technologies and human resource use [24,25]. This implies that the lower expenditures derived from the reduced ICU stay will likely also compensate for the cost of the therapy in other healthcare contexts. This could result in savings for both budget-funded providers, with neither device- nor procedure-specific reimbursement, as well as for those healthcare contexts with dedicated therapy reimbursement. Considering the latter, savings on a hospital level could be even higher.

The intraoperative use of CytoSorb^®^ therapy in IE patients has potentially important clinical and economic effects that go beyond the perioperative time investigated in this analysis. The recent literature on infective endocarditis patients observed a reduced need for vasoactive drugs in CytoSorb^®^ patients compared to control patients [12,13], as well as a diminished incidence of post-operative sepsis and septic complications [7]. Data also suggest a benefit when continuing postoperatively with hemoadsorption treatment in patients with infective endocarditis who develop renal failure in combination with severe hemodynamic instability [10]. These findings have not been captured in this analysis but might have relevant consequences in terms of patients’ morbidity and mortality, as well as on the clinical management, hospital, and societal costs associated with this population. Notably, a reduced ICU stay itself is associated with improvements in patients’ quality of life and overall health outcomes [26,27], suggesting that not only purely financial effects should be considered when appraising the value and benefits of the therapy.

Regarding the use of anti-infective therapy with hemoadsorption in IE patients, it must be acknowledged that CytoSorb^®^ therapy, along with other extracorporeal therapies, can reduce levels of vancomycin from the blood. Recently published studies recommend a 50% increase in the vancomycin dose when treating patients with concomitant hemoadsorption therapy [28,29]. Additional costs derived from potential antibiotic therapy adjustment, including medicines and drug monitoring costs, were not included in this model. The reason for their exclusion is the neglectable impact of such costs on overall ICU costs, and on the overall cost of the therapy (i.e., one unit of 500 mg of Vancomycin Hydrochloride Injection IP, 500,000 Iu/Vial costs EUR 12 to EUR 25 in Germany, depending on the package [30]).

The analysis presented in this study has some limitations. Firstly, only the costs associated with the therapy itself and to the post-operative ICU stay were considered. Other factors (i.e., antibiotic therapy adjustment, hospital length of stay, etc.) have not been incorporated in this study and might have an impact on the final calculated savings. A second limitation concerns the fact that there is uncertainty regarding the favorable effect on ICU length of stay assumed and modeled in the study. Finally, data on ICU occupation in IE patients receiving intraoperative hemoadsorption are mixed and, although reporting improvements in patients’ outcomes, recent studies have not confirmed the reduction in ICU length of stay observed in the study used to inform this model, suggesting that further data are needed to confirm the validity of this model in a real-world setting [7,14,15,16,17].

## 5. Conclusions

Our budget impact model could show that the intraoperative use of adjunctive hemoadsorption with CytoSorb^®^ in infective endocarditis patients has the potential to lead to important cost savings. However, these findings must be confirmed by further prospective analyses reporting benefits in terms of reduced intensive care unit stay.

## Figures and Tables

**Table 1:** Cost input of the budget impact model.

Item	Costs (€)	Source
CytoSorb^®^ CPB Kit for integration in the CPB or ECMO circuit (incl. 19% VAT)	1284	CytoSorbents Europe GmbH, 2023 price list
Personnel costs (for priming, installation, control, and removal of the adsorber)	60	Medical Controlling Department, University of Essen
Infrastructure surcharge (+12%)	160	
Total cost of CytoSorb^®^ therapy	1505	
ICU stay, daily cost	1640	Martin et al., 2008 [20]

CPB: cardiopulmonary bypass, ECMO: extracorporeal membrane oxygenation, ICU: Intensive care unit.

## Data Availability

No new clinical data were created or analyzed in this study. The economic data and results presented in this study are available in the Supplementary material (Microsoft Excel budget impact model).

## Supplementary Materials

The following supporting information can be downloaded at https://www.mdpi.com/article/10.3390/jcdd10090366/s1.

## Appendix A

All surgical diagnostic-related groups (DRGs), reporting both the OPS procedure code 8-821.2 ‘Extracorporeal adsorption of low and medium sized molecular, hydrophobic substances (including cytokine adsorption)’ and a primary or secondary diagnosis code for infective endocarditis (I33.0 and I33.9), were selected. By searching the database of the reimbursement.INFO platform [21], we identified a total of 2489 infective endocarditis patients who underwent heart valve surgery with a heart lung machine (cases grouped in DRGs from F03A to F03D). In a second step, we searched through all episodes and DRGs where the OPS procedure code 8-821.2 ‘Extracorporeal adsorption of low and medium sized molecular, hydrophobic substances (including cytokine adsorption)’ was used. In total, 16,720 coded adsorption treatments were identified and coded throughout 94 medical and surgical DRGs of various complexity. We combined data on the coding of the hemoadsorption procedure with data on the four surgical DRGs for IE patents requiring heart valve surgery to select 634 cases where the procedure code 8-821.2 was used. There were no other relevant surgical DRGs where the diagnosis codes for infective endocarditis were used in combination with the procedure code for hemoadsorption.

To account for possible overestimates due to the use of the therapy in the post-operative setting only or in the coding of other devices, the value was rounded down to 550 patients.

**Table A1:** Surgical DRGs for IE patients requiring cardiac surgery and use of hemoadsorption procedure.

		IE as Primary Diagnosis		IE as Secondary Diagnosis		Total	OPS Code 8.821.2	
DRG Code	DRG Label	*n*	*%*	*n*	*%*	*n*	*n*	*%*
F03D	Heart valve surgery with HLM, age > 0 years. Intensive care complex treatment < 197/185/- points, with double surgery or congenital heart defect, without complex surgery or age < 16 years or without double surgery, except congenital heart defect, age >15 years with implanted valve-bearing vascular prosthesis	588	9.2	594	9.29	1182	178	1.32
F03A	Heart valve surgery with HLM, with certain complicating constellations or certain double surgery	240	3.75	268	4.19	508	216	1.6
F03C	Heart valve surgery with HLM, age > 0 years. Intensive care complex treatment > 196/184/- points and intensive care complex treatment < 393/369/- points with double surgery or congenital heart defect, with complex surgery or pre-existing heart valve surgery or other complicating constellation	227	3.55	230	3.6	457	130	0.96
F03B	Heart valve surgery with heart-lung machine, with multiple surgeries or age < 1 year or surgery in deep hypothermia or intensive care complex treatment > 392/368/- points or certain other complicating constellation or pulmonary endarterectomy	161	2.52	181	2.83	342	110	0.81
